# The importance of selecting a cruciate-retaining total knee prosthesis for a patient with a large physiological posterior tibial slope: a case report

**DOI:** 10.1093/jscr/rjac584

**Published:** 2022-12-20

**Authors:** Ayakane Yamamoto, Takao Kaneko, Kazutaka Takada, Shu Yoshizawa

**Affiliations:** Ichinomiya Onsen Hot Hospital, Adult Reconstruction Center, 1745 Tsuboi, Ichinomiyacho, Fuefuki-city, Yamanashi 405-0077, Japan; Ichinomiya Onsen Hot Hospital, Adult Reconstruction Center, 1745 Tsuboi, Ichinomiyacho, Fuefuki-city, Yamanashi 405-0077, Japan; Ichinomiya Onsen Hot Hospital, Adult Reconstruction Center, 1745 Tsuboi, Ichinomiyacho, Fuefuki-city, Yamanashi 405-0077, Japan; Ichinomiya Onsen Hot Hospital, Adult Reconstruction Center, 1745 Tsuboi, Ichinomiyacho, Fuefuki-city, Yamanashi 405-0077, Japan

**Keywords:** cruciate-retaining total knee arthroplasty, Vanguard cruciate retaining system, posterior tibial slope, ultra-high-molecular-weight polyethylene

## Abstract

For Japanese individuals, deep bending is inevitable in their daily lives, such as during seiza sitting and kneeling. Thus, achieving a good post-operative range of motion is an important factor in improving patient satisfaction. Even normal knees often have a posterior tibial slope of more than 10°. We report the case of a 76-year-old woman who underwent proximal tibial osteotomy at 8° with the Vanguard Knee cruciate retaining total knee arthroplasty (TKA) system. She required the revision TKA 10 years later due to ultra-high-molecular-weight polyethylene wear and breakage of the posteromedial tibial component.

## INTRODUCTION

Total knee arthroplasty (TKA) is one of the most successful treatments for reducing pain and improving function in patients with end-stage knee osteoarthritis. Several national registries with long-term follow-up data show that TKA implants have a survival rate of over 90% at 10 years [1–4]. However, 15–20% of patients are not satisfied with their new joint, and up to one-third of patients report that their joint does not feel normal after TKA [5, 6]. In Japan, other Asian countries, and countries in the Middle East, achievement of very deep flexion (such as seiza sitting, sitting cross-legged and kneeling) after TKA can be a critical component of patient satisfaction for individuals who are used to a floor-sitting lifestyle. Therefore, to obtain a deep flexion angle after surgery, it is essential to maintain the joint line, reproduce the patient’s original posterior tibial slope and select a total knee prosthesis.

We report a case of TKA involving a cruciate-retaining (CR) prosthesis in a patient with a large physiological posterior tibial slope who underwent revision TKA 10 years after surgery due to ultra-high-molecular-weight polyethylene (UHMWPE) wear and breakage of the posteromedial tibial component.

## CASE PRESENTATION

A 76-year-old woman with a body mass index of 23.8 kg/m^2^ was referred to our hospital with a chief complaint of worsening right knee pain and instability (varus instability) for 1 year. At age 66, primary right TKA was performed for right knee osteoarthritis (OA) at another hospital with the Vanguard Complete Knee System CR (VCR; Zimmer Biomet, Warsaw, IN, USA). The active range of motion (ROM) in the right knee ranged from 0 to 100° with no extension. The preoperative 1989 Knee Society knee and function scores [7] were 15 and 50 points, respectively.

**Figure 1:**
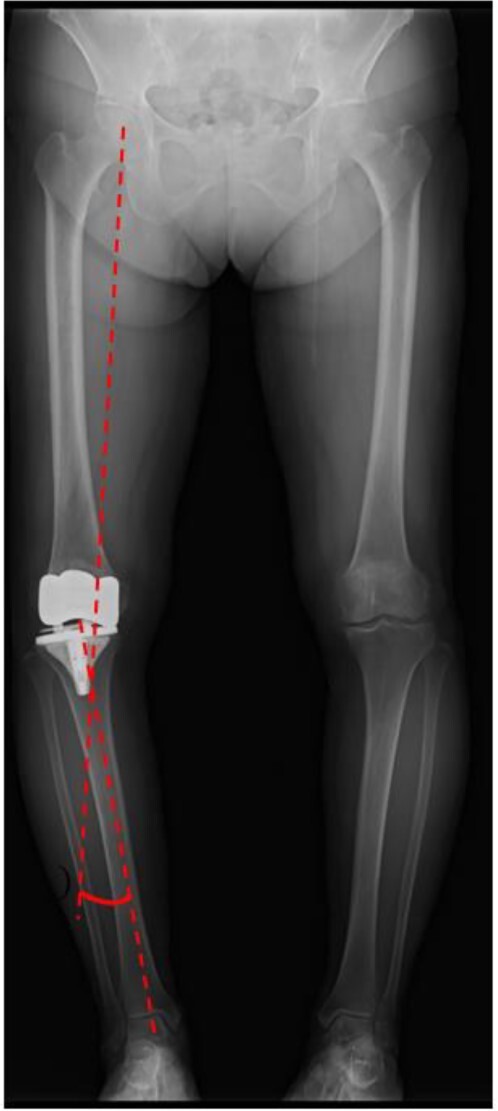
Post-operative standing plain radiograph. The hip-knee-ankle angle was 14° varus.

**Figure 2:**
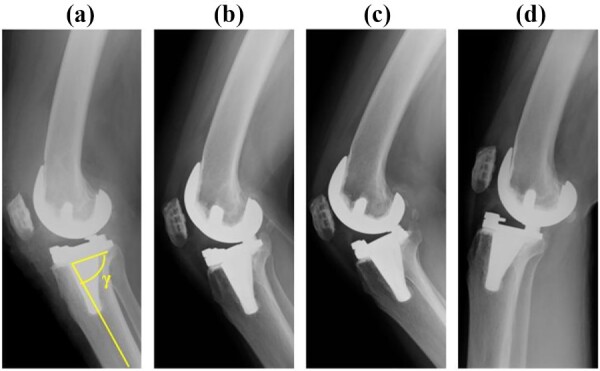
Post-operative anteroposterior and lateral plain radiographs. The γ angle was 82°. (**a**) Immediate after surgery, (**b**) 5 years after surgery, (**c**) 8 years after surgery, (**d**) 10 years after surgery.

**Figure 3:**
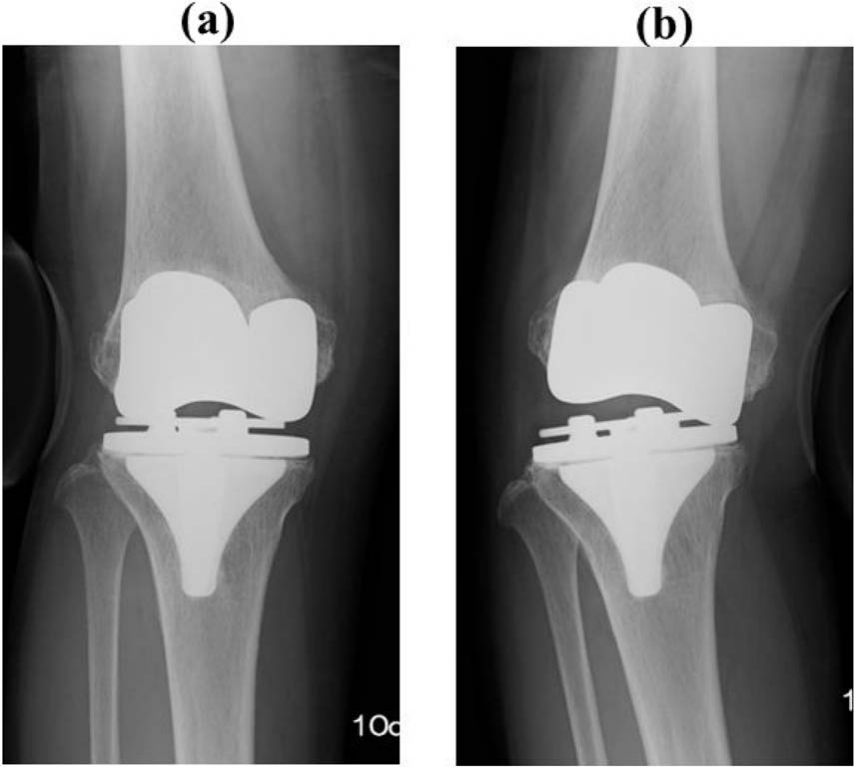
Post-operative varus and valgus stability of the knee joint in extension were assessed with stress radiography using a Telos arthrometer (Telos SD 900 Stress Device; Telos Medical Co., Ltd, USA). The patient was instructed to lie supine on a table for the measurement. During varus and valgus stress testing, a force of 150 N was applied just above the joint on the lateral or medial femoral condyle [8]. The patient had varus instability on the (**a**) valgus stress test and the (**b**) varus stress test.

**Figure 4:**
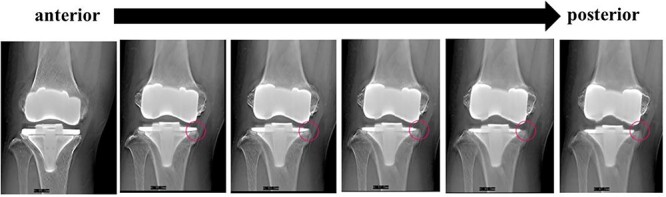
Cross-sectional anteroposterior imaging using Tomosynthesis-Shimadzu metal artifact reduction technology (T-smart) (Japan) showed tibial component subsidence at the medial tibial plateau (red circles).

A preoperative standing anteroposterior plain radiograph of the right knee showed a hip-knee-ankle angle of 14 varus alignment (Fig. 1). Preoperative lateral plain radiographs showed posterior subluxation of the femoral component at 10 years after surgery (Fig. 2). Preoperative varus and valgus stability of the knee joint in extension were assessed with stress radiography using a Telos arthrometer (Telos SD 900 Stress Device; Telos Medical Co., Ltd, USA). The patient was instructed to lie supine on a table for the measurement. During varus and valgus stress testing, a force of 150 N was applied just above the joint on the lateral or medial femoral condyle to test for varus or valgus instability (Fig. 3) [8]. Cross-sectional anteroposterior imaging using Tomosynthesis-Shimadzu metal artifact reduction technology (T-smart) (Japan) showed tibial component subsidence at the medial tibial plateau (Fig. 4).

**Figure 5:**
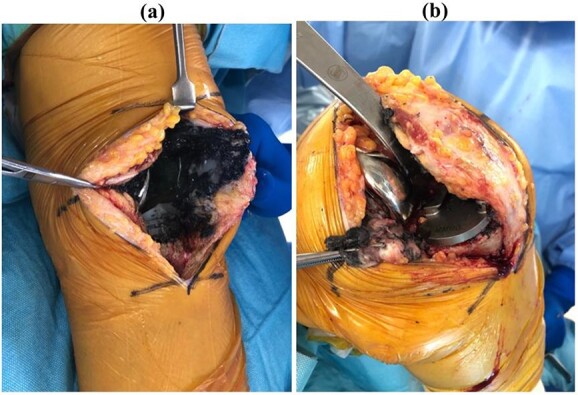
Intraoperative photograph in (**a**) extension and (**b**) flexion. Black deposits associated with metallosis were observed throughout the intra-articular joint.

**Figure 6:**
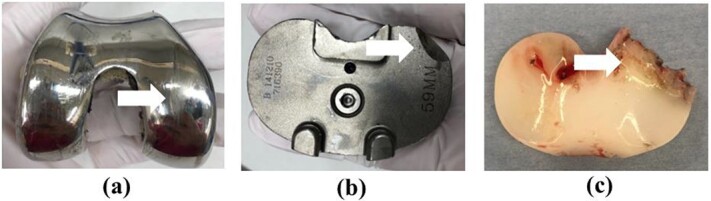
Images of the removed prosthesis. (**a**) Wear of the medial part of the femoral component (white arrow). (**b**) Breakage of the posteromedial part of the tibial component (white arrow). (**c**) UHMWPE wear (white arrow).

Revision TKA using a medial sub-vastus approach was performed under general anesthesia. Black deposits associated with metallosis were observed throughout the intra-articular joint (Fig. 5). Once the VCR prostheses are removed, wear of the medial part of the femoral component and UHMWPE wear were observed in an area consistent with breakage of the posteromedial portion of the tibial component (Fig. 6).

**Figure 7:**
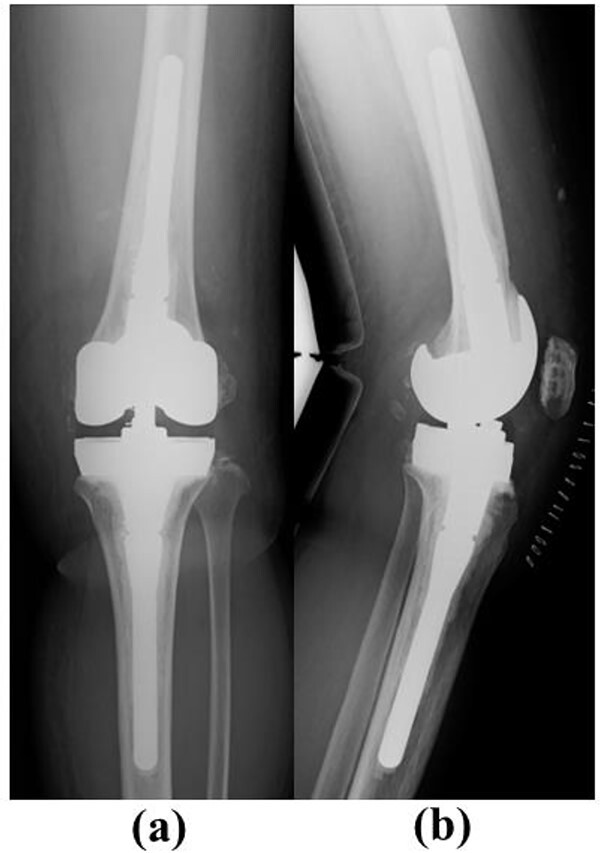
Post-operative plain radiographs after revision total knee arthroplasty. (**a**) Anteroposterior view and (**b**) lateral view. The NexGen LCCK (Zimmer Biomet, Warsaw, IN, USA) with stem extension on both the femoral and tibial sides was chosen. Full block augmentation (10 mm) was used on the tibial side.

The NexGen Legacy Constrained Condylar Knee (LCCK) (Zimmer Biomet) with stem extension on both the femoral and tibial sides was chosen. Full block augmentation (10 mm) was used on the tibial side (Fig. 7).

At 1 year after revision surgery, active ROM improved from 0 to 120°. The 2011 Knee Society Score symptom, patient satisfaction, patient expectation and daily activity subscores [9] were 24, 30, 9 and 92, respectively, compared with 5, 10, 15 and 55 before revision surgery.

## DISCUSSION

We performed revision TKA due to UHMWPE wear at a site consistent with breakage of the posteromedial portion of the tibial component at 10 years after primary VCR prosthesis placement. Varus instability was demonstrated in a varus stress test.

Tibial alignment in the sagittal plane is very important because it affects everything from durability to kinematics, such as loosening and wear. In a CR prosthesis, a larger posterior tibial slope facilitates posterior translation of the femoral component in flexion, which is advantageous for ROM acquisition. In addition, it is desirable to osteotomize the tibia at the preoperative posterior tibial tilt slope in terms to maintain bone quality at the osteotomy surface. It has been reported that the tibial component sinks less when the post-operative posterior tibial slope is closer to the preoperative value [10]. PCL tension changes when the posterior tibial slope changes after surgery [11]. Seo *et al*. reported good clinical results when the pre- and post-operative posterior tibial slopes are equal [12].

Japanese individuals have a large physiologic posterior tibial slope; the mean posterior tibial slope at the medial plateau is 10.7° (range, 5–15.5°) in normal knees [13]. Deep bending is an essential part of the Japanese lifestyle (e.g. seiza sitting or kneeling). Matsuda *et al*. revealed that post-operative alignment and ROM affect patient satisfaction after TKA [14]. There are reports of increased UHMWPE wear when the posterior tibial slope is greater than 10° [15]. When a posterior-stabilised prosthesis is selected, the flexion gap will be larger because the posterior cruciate ligament is resected [16]. Therefore, it is better to reduce the posterior tibial slope to maintain the flexion gap. In addition, if the femoral component is placed perpendicular to the distal anatomical axis, it will be in a slightly more flexed position than the functional axis, so anterior impingement of the post can be avoided if the tibial component does not have an excessive posterior tibial slope [17].

In a study based on three-dimensional computed tomography, Yamagami *et al*. found that using a posterior tibial slope of more than 9° can damage the insertion of the semimembranosus tendon, resulting in rotatory instability [18]. In this patient, the tibial osteotomy was performed with a posterior tibial slope of 8° (Fig. 2) and UHMWPE (standard CR type) with a posterior slope of 3° was selected. The resulting 11° of posterior tilt resulted in UHMWPE wear and breakage of the posteromedial tibial baseplate at 10 years. In addition to the Standard CR, the Vanguard CR system also includes the CR Lipped and Anterior Stabilised types, which have flat articular surfaces. For patients with a large physiologic posterior tibial tilt, a standard CR prosthesis with a built-in posterior tilt of 3° should not be selected.

The VCR was introduced in 2003. Montonen *et al*. compared survival rates of several CR prostheses: Triathlon CR (Stryker, Mahwah, NJ, USA), NexGen CR Flex (Zimmer Biomet), PFC Sigma CR (DePuy, Warsaw, IN, USA) and VCR. They found that the VCR system is associated with a significantly greater risk of revision surgery than the NexGen CR Flex system [19].

Crawford *et al*. followed 1664 knees after primary TKA with the VCR system for a minimum of 10 years. It had excellent long-term survival, at 95.2% with the revision rate as the endpoint [20]. On the other hand, the Swedish Knee Arthroplasty Registry reported that the VCR system had a significantly higher relative risk of revision surgery than other prostheses [21]. In addition to the VCR system, it should also be noted that there is a LEGION Primary Knee CR System (Smith & Nephew, Andover, Texas, USA) that has a posterior tibial slope of 4° with UHMWPE.

The thickness of the tibial component of each prosthesis is shown in Table 1. The VCR system has a thicker tibial component (4.0 mm) than other prostheses. However, breakage of the tibial component occurred because the femoral component moved posteriorly due to the function of the posterior cruciate ligament in flexion, resulting in high levels of physical loading.

**Table 1:** Differences in tibial component thickness

**Prosthesis**	**Thickness (mm)**
NexGen CR & PS FLEX (Zimmer Biomet)	3.5
PERSONA CR & PS FLEX (Zimmer Biomet)	3.5
GENESIS CR & PS (Smith & Nephew)	2.3
LESION CR & PS (Smith & Nephew)	2.3
Journey II BCS & CR (Smith & Nephew)	2.3
Vanguard CR & PS (Smith & Nephew)	4.0
Attune CR & PS (Smith & Nephew)	4.0
Scorpio NRG CR & PS (Stryker)	2.0
Triathlon CR & PS (Stryker)	3.0

In conclusion, if the posterior tibial slope is physiologically large and a VCR system is selected, the CR Lipped and Anterior Stabilised type is preferred over the standard CR type. Surgeons should be familiar with the prosthetic design.

## CONFLICT OF INTEREST STATEMENT

The authors, their immediate family, and any research foundation with which they are affiliated did not receive any financial payments or other benefits from any commercial entity related to the subject of this article.
